# The moderating effect of childhood disadvantage on the associations between smoking and occupational exposure and lung function; a cross sectional analysis of the UK Household Longitudinal Study (UKHLS)

**DOI:** 10.1186/s12889-019-7039-z

**Published:** 2019-06-04

**Authors:** Caroline Carney, Michaela Benzeval

**Affiliations:** 1 Institute for Social and Economic Research (ISER), University of Essex, Wivenhoe Park, Colchester, CO4 3SQ UK; 2 LSE Health, London School of Economics and Political Science (LSE), Cowdray House, Houghton Street, London, WC2A 2AE UK

**Keywords:** Lung function, Socio-economic position, Health behaviours, Occupational exposures, UKHLS, Cross-sectional

## Abstract

**Background:**

Lung function is lower in people with disadvantaged socio-economic position (SEP) and is associated with hazardous health behaviours and exposures. The associations are likely to be interactive, for example, exposure to socially patterned environmental tobacco smoke (ETS) in childhood is associated with an increased effect of smoking in adulthood. We hypothesise that disadvantaged childhood SEP increases susceptibility to the effects of hazards in adulthood for lung function. We test whether disadvantaged childhood SEP moderates smoking, physical activity, obesity, occupational exposures, ETS and air pollution’s associations with lung function.

**Methods:**

Data are from the Nurse Health Assessment (NHA) in waves two and three of the United Kingdom Household Longitudinal Study (UKHLS). Analysis is restricted to English residents aged at least 20 for women and 25 for men, producing a study population of 16,339. Lung function is measured with forced expiratory volume in the first second (FEV_1_) and standardised to the percentage of expected FEV_1_ for a healthy non-smoker of equivalent age, gender, height and ethnicity (FEV_1_%). Using STATA 14, a mixed linear model was fitted with interaction terms between childhood SEP and health behaviours and occupational exposures. Cross level interactions tested whether childhood SEP moderated household ETS and neighbourhood air pollution’s associations with FEV_1_%.

**Results:**

SEP, smoking, physical activity, obesity, occupational exposures and air pollution were associated with lung function. Interaction terms indicated a significantly stronger negative association between disadvantaged childhood SEP and currently smoking (coefficient -6.47 %, 95% confidence intervals (CI): 9.51 %, 3.42 %) as well as with formerly smoking and occupational exposures. Significant interactions were not found with physical activity, obesity, ETS and air pollution.

**Conclusion:**

The findings suggest that disadvantaged SEP in childhood may make people’s lung function more susceptible to the negative effects of smoking and occupational exposures in adulthood. This is important as those most likely to encounter these exposures are at greater risk to their effects. Policy to alleviate this inequality requires intervention in health behaviours through public health campaigns and in occupational health via health and safety legislation.

## Background

Lung function is known to be lower in adults who had disadvantaged socio-economic position (SEP) in their childhood [1–4]. Lung function indicates how well the lungs bring oxygen into the blood, remove carbon dioxide and how strong breathing muscles are, weak lung function impairs the body’s ability to get the oxygen it needs to create energy. Evidence suggests the environment in utero may be important for lung function both in infancy and in later life. Birth weight, which is socially patterned [5] is indicative of in utero environment and is associated with adult lung function [6, 7]. Socially patterned childhood factors including birth weight, breast-feeding and lower tract respiratory infections (LTRI) were significant predictors of adult lung function in research using the Newcastle Thousand Families Study [8], indicating the continued importance of childhood exposures for adult lung function. The authors suggested their findings support the fetal programming hypothesis whereby impaired development in utero results in increased risk of poor health in adulthood. Exposures such as maternal smoking and diet when in utero and environmental tobacco smoke (ETS) in childhood are socially patterned and associated with lung function in childhood and in adulthood [9–11]. Research on the 1958 National Childhood Development Study found financial adversity in childhood was related to midlife lung function and the association was mediated by housing deprivation, continuation of disadvantage and smoking in adulthood [4].

Childhood experience has long-term implications for respiratory health through influencing the growth and development of the lungs. Childhood disadvantage could additionally influence adult respiratory health by creating biological vulnerability to the effects of environmental hazards and behavioural risks for lung function. One study that found evidence of an interaction between maternal smoking and personal smoking for adult lung function suggested this resulted from a biological interaction due to their combined effect [12]. Another study of young adults found smoking was only associated with steeper lung function decline in smokers exposed to parental smoking [13]. Given evidence that suggests moderation of smoking’s effects on lung function by socially patterned exposures in childhood [12, 13], it is possible that similar modification exists with other hazardous exposures to lung function in adulthood that have similar biological pathways. Smoking impacts on lung function by causing oxidative stress and inflammation in the lungs [14]. It is likely that exposure to ETS in adulthood would influence the lungs through the same mechanism as smoking while occupational exposures and air pollution also cause oxidative stress and inflammation in the lungs [15–17]. Other risks for lung function include low physical activity [18] and obesity [19, 20]. These may also be moderated by childhood circumstances as both are related to inflammation, physical activity has been posited to protect from lung function decline through its anti-inflammatory effects [21] while obesity causes increased systemic inflammation [22]. Due to the similar pathways between ETS, occupational exposures, physical activity and obesity’s associations with lung function and smoking’s association with lung function, we hypothesise that they may also be modified by childhood experience in the same way that the association with smoking is.

Adapting a life course perspective, we hypothesise that childhood is a sensitive period for lung function whereby in utero and childhood exposures affect development and are influential for adult lung function but do not entirely determine it. We hypothesise that the effects of socially patterned exposures in adulthood also influence lung function but that these effects may be multiplicative combined with childhood influences rather than additive, resulting in an increased risk of reduced lung function greater than their combined risk. We propose that this occurs due to increased sensitivity to the effects of socially patterned exposures in adulthood because of the negative impact on the lungs’ development in childhood of socially patterned exposures such as low birth weight, LTRI and ETS.

It is important to understand whether behavioural and environmental hazards for lung function in adulthood are moderated by childhood disadvantage. Knowing this would enable policy makers to help individuals be aware of and manage their risk of reduced lung function associated with certain health behaviours. Identifying whether those with disadvantaged childhood SEP have increased susceptibility to the effects of hazardous health behaviours and exposures is useful for policy on health inequalities; as many of these hazards are socially patterned, it could imply that those most exposed to these risks are most vulnerable to their effects, which would require corrective policy action. This is important as inequalities are known to exist in lung function [1] and weak lung function is associated with increased morbidity and mortality [23, 24]. Low lung function can indicate chronic obstructive pulmonary disorder and is associated with all-cause mortality as well as mortality from ischaemic heart disease, all cancers, lung cancer, stroke, respiratory disease [24]. This paper assesses whether smoking, physical activity, obesity, occupational exposure, ETS and air pollution are associated with adult lung function, after adjustment for adult SEP, and identifies whether each of these associations are modified by disadvantaged childhood SEP.

## Methods

### Study sample

Participation in the Nurse Health Assessment (NHA) [25] of Understanding Society: the UK Household Longitudinal Study (UKHLS) [26] was invited from adults living in Great Britain during wave two of the UKHLS for the General Population Sample (GPS) and during wave three for the British Household Panel Survey (BHPS) sample. The GPS is a stratified, clustered, equal probability sample of residential addresses throughout the UK in 2009 [27]. The BHPS began as a stratified random sample initiated in 1991 with country specific boost samples [28]. NHA interviews consisted of a nurse undertaking physical functioning measures, anthropometrics and blood samples approximately five months after the main interview, beginning in May 2010. Of the 43,747 adult, British resident members of the GPS and BHPS who gave a full interview in waves two and three respectively [29], participation in the NHA was limited to those who gave a full English language interview and were not pregnant. In the second year of wave two, selection was restricted to 81 % of primary sampling units in England to allow interviewing of the BHPS sample. Of 35,937 eligible to participate in the NHA, 20,700 (57.6 %) took part [30].

### Study population

**Fig. 1:**
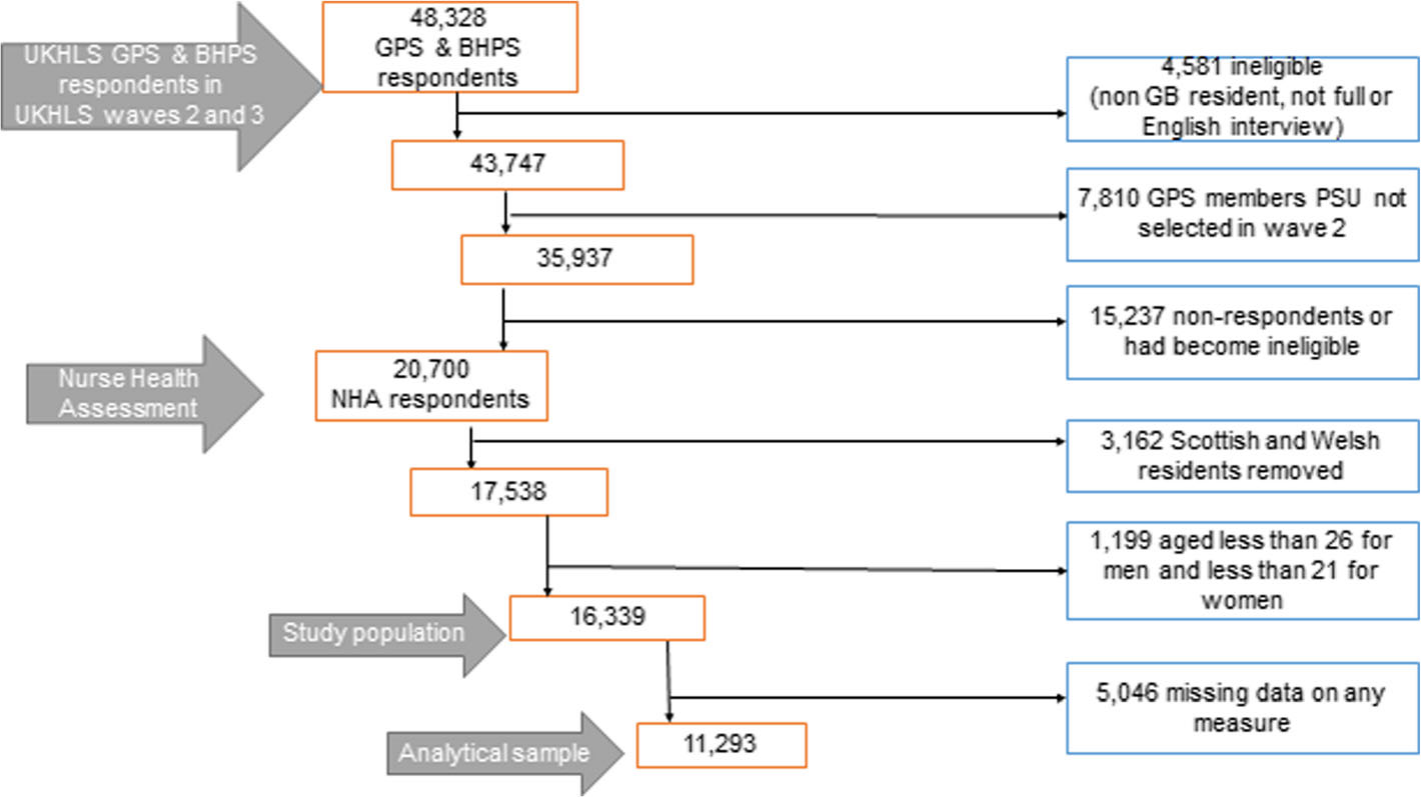
Description of restrictions applied to create study population

For the purposes of this paper, analysis was restricted to English residents as air pollution data was only consistently available for England; 1,667 Scottish and 1,495 Welsh residents were removed. Men and women younger than 26 years and 21 years respectively (1,199 cases) were removed to restrict the analysis to those whose lungs had already fully developed [31], resulting in a study population of 16,339 respondents. This is shown on Fig. 1.

### Measurement

Outcome variable - forced expiratory volume in the first second of exhalation (FEV_1_) is used to measure lung function here. FEV_1_ was captured with the electronic NDD Easy On-PC spirometer in UKHLS [29]. Normal levels are dependent on age, height, gender and ethnicity. FEV_1_ was transformed into a percentage of the expected FEV_1_ (FEV_1_%) for a healthy non-smoking person of equivalent age, height, gender and ethnicity using guidelines from the Global Lung Function Initiative [32]. A FEV_1_% between 80 % and 120 % is considered normal, FEV1% below 80 % is considered obstructed. FEV_1_ is the most widely used parameter to measure the mechanical properties of the lungs [33] and is more reproducible than forced vital capacity (FVC) [34]. FEV_1_/FVC was not considered a useful measure for the purposes of this analysis as if both FVC and FEV_1_ are reduced as in restrictive lung diseases and lung defects then a normal FEV_1_/FVC result is produced. Measurement requires participants to make an effort and it needs to be done correctly to produce a high quality measurement. Obtaining the highest quality measurement, Grade A, required participants to produce two highest FVC and FEV_1_ measurements within 100ml of each other and was only achieved by 51.3 % of those who provided measurement. Poor quality graded measurements were included in this analysis. FEV_1_% is used here as a continuous variable.

Exposures of interest – there are two key exposures of interest here smoking and occupational exposure to hazards for lung function. Respondents were asked if they had ever smoked a cigarette, pipe or cigar and those who responded positively were asked if they ever did so nowadays, based on their responses to this they were classified as current, former and never smokers. A job exposure matrix assessing risk for chronic obstructive pulmonary disorder (COPD) measured occupational exposures to dusts, gases or fumes [35]. It was linked to the SOC2000 classification of occupations and derived into whether participants were exposed to COPD risks in their current or last (if not employed) occupation. Those who were students, long term ill or disabled and carers were classified as unexposed.

Moderator – the key moderator is childhood SEP, this is in part because direct measures of socially patterned exposures, such as maternal smoking and ETS, were not available nor was birth weight which might more closely indicate the environment in utero. Maternal education was used, therefore, as a marker for such exposures by measuring childhood SEP. This was prioritised over paternal or household SEP as it was hypothesised that maternal SEP would capture the experiences in utero and in early childhood that are consequential for lung function, better than other childhood SEP measures. Paternal or household SEP may not reflect the resources available to women, important for this hypothesis, due to unequal sharing of resources within the household, which is more likely to impact negatively on women. There is a strong correlation between maternal and paternal SEP, but more missingness in the measure for fathers. Maternal occupation was asked in reference to when the respondent was aged 14 and thus may not reflect the period around birth posited here to influence lung function. Responses were derived into a dichotomous variable with categories of ‘no schooling or qualifications’ indicating disadvantaged childhood SEP, ‘some qualifications or post school qualifications indicating advantaged childhood SEP’ and ‘do not know’ (n=1,426) and ‘other’ (n=41) were classified as missing. Maternal education was asked of some GPS respondents in wave one and others in wave two, while some members of the BHPS sample responded to maternal education in wave 13 of BHPS. Responses obtained to maternal education in the three different waves were combined into one variable indicating advantaged or disadvantaged childhood SEP.

Confounders – a number of key confounders – age, sex, ethnicity, height and weight are not included in the models as these factors have already been included in the standardisation of lung function (see above). There are three additional confounders at the individual level – adult SEP, physical activity and obesity – as well as measures of household and area deprivation and pollution. As the exposures of interest, smoking and occupational hazards, are socially patterned and associated with other health behaviours that influence lung function; these were included in the analysis to prevent over estimation of their association. All individual level covariates were captured in wave two except waist circumference, which was captured in wave three for the BHPS sample. Educational attainment indicated adult SEP, the derived measure of highest qualification was grouped into ‘A level and higher’ indicating advantaged SEP and ‘GCSE and lower’ indicating disadvantaged. For physical activity, respondents were divided into those who participated in mild or moderate physical activity at least once a week or less than this. Waist circumference was used here to indicate obesity, very high waist circumference is defined as 102 centimetres or greater for a man and 88 centimetres or greater for a woman [36]. Measurement and exclusions from the waist measurement are described elsewhere [29]. Whether respondents were GPS or BHPS members was included as a covariate due to the different time lags in the collection of confounders.

ETS was indicated by whether the household contained a smoker. As ETS is socially patterned, household tenure was used as a proxy for household SEP. When tenure was rented from a Local Authority or Housing Association, it was classified as disadvantaged household SEP, all other tenure indicated advantaged SEP. Nitrogen dioxide, particulate matter, sulphur dioxide and benzene were captured by the ‘living environment’ domain of the 2010 Index of Multiple Deprivation (IMD). The IMD is derived for Lower Area Super Output Areas (LSOA). LSOA have varying sizes with populations between 1,000 and 3,000 individuals or 400 and 1,200 households. We use air pollution captured by the IMD for LSOA, which we linked to households LSOA in UKHLS [26]. Modelled estimates of each pollutant obtained on a 1 kilometre grid were related to a standard value defined as a risk to health or ecosystems and then summed to create an overall measure [37]. This was derived into a binary measure of whether an area was above or below the mean level of air pollution. The income domain of the 2010 IMD was included to control for area deprivation. A binary measurement of whether an area was above or below the mean level of income deprivation was created to indicate area deprivation here. More information on the measurement of air pollution and income deprivation is provided elsewhere [37].

### Statistical method

A mixed linear regression model, with three levels (individual, household and area) with main effects for each exposure of interest was fitted and then extended to include interaction effects between childhood SEP and smoking, physical activity, obesity and occupational exposures. As ETS and air pollution were measured within households and areas respectively, cross level interactions with childhood SEP were estimated. Random intercepts captured variation in lung function at the area and household levels but the coefficients for each parameter were assumed to have the same association across households and areas [38]. Significant interactions indicate that the association between the exposure or health behaviour and lung function was different for those with disadvantaged childhood SEP compared to those from an advantaged background.

Analysis was undertaken using Stata 14 [39]. Complete case analysis was employed without any imputation of missing values. This approach was taken because missingness was mainly the result of two key variables. Non-response was high on the FEV_1_ and was unlikely to be random as poor lung function can affect the ability to provide lung function measurement, and on maternal education. Multiple imputation is an effective approach to addressing missingness when it is spread across a range of variables. Complete case analysis produces unbiased estimates if the outcome is not associated with being a complete case once confounders are controlled for [40]. We estimated whether the outcome was associated with being a complete case using logistic regression with control for confounders, as it was not, complete case analysis is used here (full results available on request). The profile of the study population was compared to the analytical sample (those without non-response on any measure) in the presentation of descriptive results. Given high levels of missingess on the outcome variable, we carried out a sensitivity test^1^. We reran the models incorporating those without lung function measurement as having low FEV_1_ to assess if estimates were consistent with the main analysis. As an additional sensitivity test, the analysis was rerun separately for those with a ‘Grade A’ quality lung function measurement and all other levels of quality. The results to both reflect those presented here and hence are not included but are available on request.

The cross sectional weight for the combined NHA sample was used throughout to adjust for unequal selection probabilities and differential nonresponse to the NHA.

## Results

**Fig. 2:**
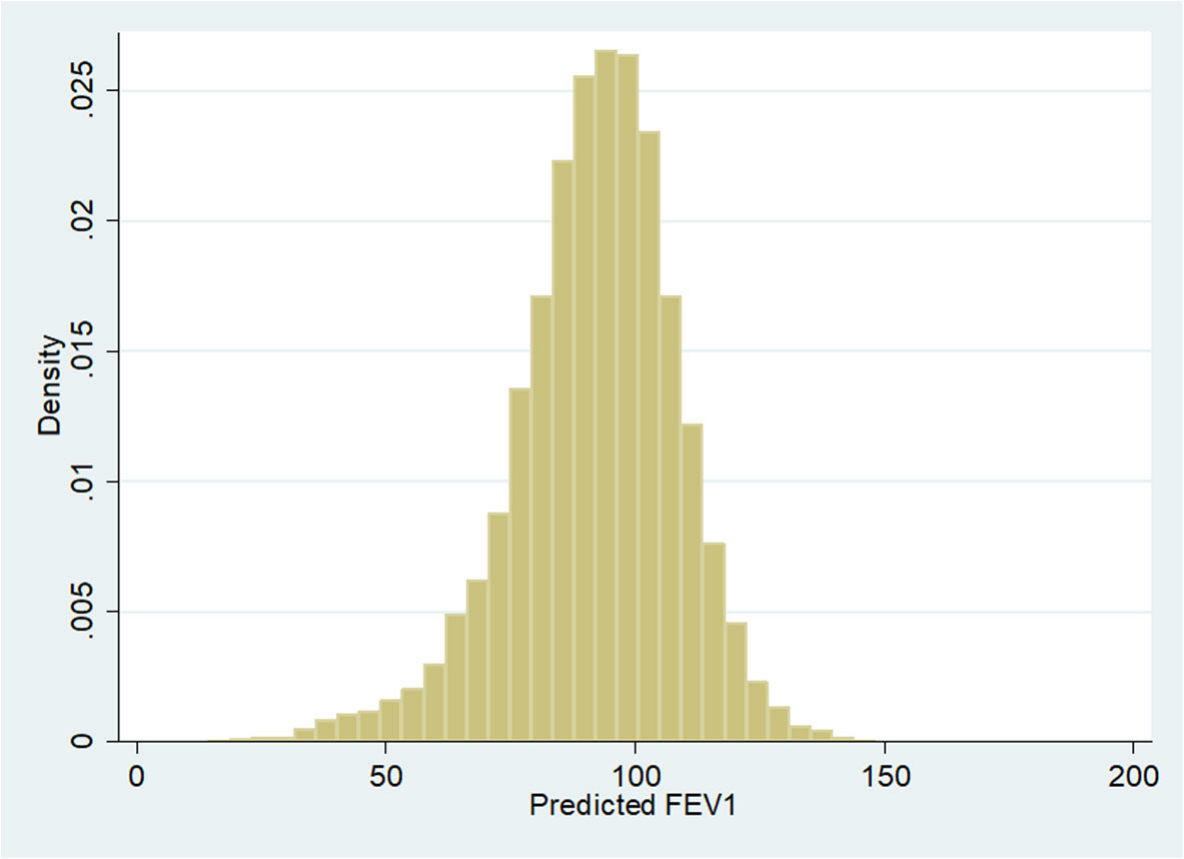
Distribution of FEV_1_%

Figure 2 shows the distribution of FEV_1_% in the analytical sample, the mean was 92.28 % with a standard deviation (SD) of 16.39. The median was 93.10 %, the distribution had a slight negative skew.

There were 5,046 members of the study population with non-response to at least one measure. This was mainly due to non-response to FEV_1_%, 2,255 non-respondents, and to maternal education, which had 2,090 non-respondents. For FEV_1_, there were 947 not eligible for measurement due to reasons such as pregnancy, having had abdominal or chest surgery in the last three weeks, being hospitalised with a heart complaint in the last six weeks, having an eye surgery in the last four weeks or having a tracheostomy. A further 307 refused to provide measurement. Table 1 shows the mean and standard deviation (SD) of FEV_1_% in the study population and the analytical sample as well as their profile in regards to exposures and covariates. Mean FEV_1_% in the study population was 91.98 % (SD 16.48), it was slightly higher in the analytical sample at 92.28 % (SD 16.39). The analytical sample was younger, and reported slightly poorer health behaviours. A larger proportion in the analytical sample reported disadvantaged childhood SEP than in the full study sample. However smaller proportions of the analytical sample reported disadvantaged adult SEP and household SEP. Comparing the profiles of the study population and analytical samples implies that more of those in poor health and with disadvantaged SEP were excluded from the analytical sample which suggests analysis may underestimate the true association in the population.

**Table 1:** Descriptive characteristics for study population and analytical sample

	Study population (*n*=16,339)	Analytical sample (*n*=11,293)
Lung function		
Mean FEV_1_% (SD)	91.98 % (16.48)	92.37 % (16.12)
Missing	13.40 %	0.00 %
Demographics		
Gender		
Female	56.93 %	56.50 %
Missing	0.00 %	0.00 %
Age		
Mean age (SD)	50.54 (17.20)	48.67 (16.12)
SEP		
Childhood SEP		
Disadvantaged childhood SEP	42.67 %	45.18 %
Missing	12.24 %	0.00 %
Adult SEP		
Disadvantaged adult SEP	46.53 %	41.26%
Missing	0.22 %	0.00 %
Health behaviours and exposures		
Smoking		
Smoker	20.43 %	20.01 %
Former smoker	38.89 %	38.09 %
Missing	0.08 %	0.00 %
Physical activity		
Low physical activity	39.83 %	36.63 %
Missing	0.71 %	0.00 %
Obesity		
Obese	41.14 %	42.48 %
Missing	1.90 %	0.00 %
Occupational exposures		
Exposure to COPD hazards	38.82 %	40.01 %
Missing	6.27 %	0.00 %
Household		
Household smoking		
ETS	26.78 %	26.35 %
Missing	0.01 %	0.00 %
Household SEP		
Disadvantaged household SEP	17.02 %	14.48 %
Missing	0.21 %	0.00 %
Area		
Air pollution		
Above average air pollution	37.64 %	38.21 %
Missing	0.12 %	0.00 %
Area deprivation		
Above average area deprivation	37.56 %	36.21 %
Missing	0.12 %	0.00 %

The analytical sample was 56.50 % female with a mean age of 48.67 (SD 16.12). Disadvantaged SEP in childhood and adulthood was reported by 45.18 % and 41.26 % respectively. There were 20.01 % and 38.09 % current and former smokers respectively. More than one-third reported low physical activity, 42.48 % were obese and 40.01 % were exposed to COPD hazards. Approximately one quarter was exposed to ETS in their household and 38.07 % lived in areas with above average air pollution.

Table 2 shows the coefficients and confidence intervals (CI) from a mixed model with main effects only for each covariate of interest (model 1) and then with the addition of interaction and cross-level interaction effects (model 2). In model 1, those with disadvantaged childhood SEP had a FEV_1_% 2.52 % (3.18 %, 1.87 %) lower than those with advantaged childhood SEP. Disadvantaged adult SEP was also significantly associated with lower FEV_1_%; -1.28 % (-1.97 %, -0.59 %). In current and former smokers, FEV_1_% was 4.58 % (6.13 %, 3.03 %) and 0.98 % (1.97 %, 0.59 %) lower than never smokers respectively. Low physical activity was associated with FEV_1_% 2.83 % (3.52 %, 2.14 %) lower than in physically active respondents. Being obese was associated with having FEV_1_% 3.61 % (4.26 %, 2.97 %) lower than those of normal waist circumference. Living in a household with ETS was not associated with FEV_1_%, though disadvantaged household SEP was associated with having lower FEV_1_% producing a coefficient of -2.58 % (-3.70 %, -1.46 %). Living in an area with above average air pollution was associated with FEV_1_% being 1.66 % (2.36 %, 0.95 %) lower than those in areas with less air pollution and those in disadvantaged areas had FEV_1_% 0.96 % (1.71 %, 0.22 %) lower than those in advantaged areas.

**Table 2:** Association between SEP measures, health behaviours, environmental hazards and FEV_1_% and interactions between childhood SEP with health behaviours and environmental hazards

		Model 1	Model 2
Main effects	Value	Coefficient (95 % CI)	Coefficient (95 % CI)
Childhood SEP	Advantaged childhood SEP as reference		
	Disadvantaged childhood SEP	-2.52 % (-3.18 %, -1.87 %)	0.78 % (-0.52 %, 2.07 %)
Adult SEP	Advantaged adult SEP as reference		
	Disadvantaged adult SEP	-1.28 % (-1.97 %, -0.59 %)	-1.33 (-2.02 %, -0.64 %)
Smoking	Never smoker as reference		
	Smoker	-4.58 % (-6.13 %, -3.03 %)	-1.67 % (-3.55 %, 0.21 %)
	Former smoker	-0.98 % (-1.67 %, -0.34 %)	0.35 % (-0.51 %, 1.20 %)
Physical activity	Physically active as reference		
	Low physical activity	-2.83 % (-3.52 %, -2.14 %)	-2.15 % (-3.04 %, -1.26 %)
Obesity	Not obese as reference		
	Obese	-3.61 % (-4.26 %, -2.97 %)	-3.46 % (-4.29 %, -2.63 %)
Occupational exposure	No occupational exposures as reference		
	Occupational exposure to COPD hazards	-0.88 % (-1.54 %, -0.23 %)	-0.08 % (-0.91 %, 0.76 %)
Household			
Household smoking	No ETS as reference		
	ETS	-0.42 % (-1.78 %, 0.95 %)	-0.93 % (-2.55 %, 0.69 %)
Household SEP	Advantaged household SEP as reference		
	Disadvantaged household SEP	-2.58 % (-3.70 %, -1.46 %)	-2.51 % (-3.62 %, -1.39 %)
Area			
Air pollution	Below average air pollution as reference		
	Above average air pollution	-1.66 % (-2.36 %, -0.95 %)	-1.63 % (-2.49 %, -0.76 %)
Area deprivation	Below average area deprivation as reference		
	Above average area deprivation	-0.96 % (-1.71 %, -0.22 %)	-0.91 % (-1.66 %, -0.17 %)
Interactions			
Smoker x disadvantaged childhood SEP			-6.47 % (-9.51 %, -3.42 %)
Former smoker x disadvantaged childhood SEP			-2.71 % (-4.09 %, -1.34 %)
Low physical activity x disadvantaged childhood SEP			-1.29 % (-2.63 %, 0.05 %)
Obese x disadvantaged childhood SEP			-0.38 % (-1.68 %, 0.92 %)
Occupational exposure x disadvantaged childhood SEP			-1.55 % (-2.85 %, -0.25 %)
Cross level interactions			
ETS x disadvantaged childhood SEP			1.01 % (-1.59 %, 3.61 %)
Air pollution x disadvantaged childhood SEP			0.01 % (-1.35 %, 1.34 %)
Constant		99.68 % (98.99 %, 100.36 %)	98.16 % (97.36 %, 98.97 %)

In model 2 significant interactions were observed between childhood SEP and smoking and occupational exposures but not with physical activity, obesity, ETS or air pollution. The interaction between smoking and disadvantaged childhood SEP was associated with FEV_1_% 6.47 % (9.51 %, 3.42 %) lower, indicating that the effect of smoking in adulthood is worse for those with disadvantaged childhood SEP. Childhood SEP significantly modified formerly smoking’s association with lung function, former smokers with disadvantaged childhood SEP had FEV_1_% 2.71 % (4.09 %, 1.34 %) lower than those who never smoked and had advantaged childhood SEP. The association between formerly smoking and FEV_1_% was not significant for those with advantaged childhood SEP. Having disadvantaged childhood SEP and exposure to COPD hazards was associated with having FEV_1_% 1.55 % (2.85 %, 0.25 %) lower than for those without disadvantaged childhood SEP or occupational exposures. Similarly to the associations with smoking and formerly smoking, the association between occupational exposures and lung function was not significant for those with advantaged childhood SEP.

The main effect for disadvantaged childhood SEP was not significant once the interactions were added to the model. Low physical activity was associated with having FEV_1_% 2.15 % (3.04 %, 1.26 %) lower and being obese was associated with having FEV_1_% 3.46 % (4.29 %, 2.63 %) lower than those who were physically active and a healthy weight respectively. As in model 1, ETS did not have a significant association with FEV1% while household SEP had a significant negative association with FEV_1_%. Living in an area with above average deprivation and air pollution were both significantly associated with lower FEV_1_%.

## Discussion

### Key results

Our findings indicate sensitivity to some lung function hazards being influenced by childhood disadvantage. We found disadvantaged childhood SEP had significant interactions with current and former smoking and occupational exposures but not with physical activity, obesity, ETS or air pollution. The mechanisms through which smoking and occupational exposures as well as ETS and air pollution affect lung function are mainly oxidative stress and inflammation. Thus, it is plausible to expect that the moderating effect of childhood would be similar for each, however this was not found here. In regards to ETS this may be due to the use of a proxy measure of ETS based on whether there was a smoker present in the household but not whether they smoked indoors, how much they smoked or to the extent to which members of the household were exposed. The main effect for ETS was not significant either. The air pollution measure used was less limited though it was based on modelled estimates from 2008, which may have differed from air pollution at the time of the NHA. Additionally, it may not capture the extent to which people were exposed to air pollution in their neighbourhood, how mobile they were outside of their LSOA and the modes of transport used. Physical activity can protect against oxidative stress and inflammation, the coefficient for the interaction between low physical activity and childhood disadvantage indicated a negative trend though this was not significant nor was the interaction between childhood disadvantage and obesity. The analysis also shows adult SEP as well as household and area SEP all have independent associations with lung function.

Previous research has not tested whether childhood SEP moderates the effect of health behaviours and occupational and environmental exposures on adult lung function, however some similar research exists. Our findings support that of Guerra et al [13] who found that steeper lung function decline was only present in smokers who also reported parental smoking. We found that the main effect associated with smoking and formerly smoking attenuated once the interaction with childhood disadvantage was added though our study differs in that we explore interactions with childhood disadvantage rather than parental smoking.

Our research shows childhood SEP is important for adult lung function although this association appears to be moderated through adult behaviours and environmental exposures. Our findings suggest that childhood may be a sensitive period for lung function, which is supported by previous indications that socially patterned exposures in childhood affect lung size, alveoli size and bronchial hyper-responsiveness [10]. This could indicate a biological pathway between childhood SEP and adult lung function though other research has suggested that childhood is socially rather than biologically pertinent for adult lung function [4].

### Limitations and strengths

This study has several limitations pertaining to measurement and the sample used. Many of the measures used were operationalised as binary variables, which may oversimplify the experience of health behaviours, obesity, occupational exposure to COPD hazards, air pollution and SEP. The measure of childhood SEP, maternal education, as noted above may not adequately capture the elements of childhood and in utero environment important for lung function. Future research may benefit from considering the moderating effect of more detailed measures of socially patterned exposures in childhood such as maternal smoking, exposure to ETS or history of LTRI to better understand the pathways through which childhood SEP is associated with adult lung function. Some key confounders were also limited in their measurement. The measure of exposure to ETS was a proxy and there was no direct measurement of whether smokers smoked indoors or if participants were exposed to ETS in other locations. The air pollution measure used did not capture the extent to which people were exposed or whether they were mobile outside of their LSOA.

The analysis presented here was restricted to England and thus the results may not be representative of the wider British population. The study benefits from a large weighted, sample of the English population. Large non-response can limit generalisability, examining the socio-demographic and health profiles of non-respondents on each of these suggest that those who are disadvantaged and in poor health are more likely to be have been excluded from the analysis. However, the sensitivity test conducted where those with non-response to lung function were recoded as having low FEV_1_% produced estimates in consistent with the main analysis suggesting missing FEV_1_% did not bias results.

## Conclusions

The association of smoking and occupational exposures with poor lung function is stronger for those with disadvantaged childhood SEP than those with an affluent childhood. Due to social patterning of health behaviours and intergenerational transmission of SEP, those with disadvantaged childhood SEP are more likely to smoke and encounter occupational exposures. Policy to alleviate inequalities in lung function requires targeted intervention directed at those who are most vulnerable to promote behavioural change for smoking and to ensure sufficient protection via health and safety legislation for occupational exposures. This may be important for setting limits to lung function hazards in occupational settings or in the environment as they are often based on the population average risk rather than the risk posed to those who are more vulnerable. The continued importance of childhood SEP in adulthood suggests that childhood may be a sensitive period for lung function and interventions to alleviate socially patterned exposures in childhood may help improve respiratory health in later life.

## Data Availability

The research datasets supporting the conclusions of this article are distributed by the UK Data Archive [https://discover.ukdataservice.ac.uk/series/?sn=2000053]. The IMD data used for LSOA income deprivation and air pollution are provided by the Ministry of Housing, Communities and Local Government [https://www.gov.uk/government/statistics/english-indices-of-deprivation-2010].

## Abbreviations

BHPS: British household panel survey
CI: Confidence interval
COPD: Chronic obstructive pulmonary disorder
ETS: Environmental tobacco smoke
FEV_1_: Forced expiratory volume in the first second of exhalation
FEV_1_%: Percentage of the expected FEV1 based on age, height, gender and ethnicity
GPS: General population sample
IMD: Index of multiple deprivation
LSOA: Lower Area Super Output Area
LTRI: Lower tract respiratory infection
N: Number of observations
NHA: Nurse health assessment
SD: Standard deviation
SEP: Socio-economic position
UKHLS: United Kingdom household longitudinal study

## Footnotes

1 Full results from all sensitivity tests are available from the corresponding author.
